# International Survey on Antibiotic Prophylaxis Approaches for Solid Organ Transplant Recipients and Donors Colonized With Multidrug‐Resistant Organisms

**DOI:** 10.1111/tid.70154

**Published:** 2025-12-16

**Authors:** Julia Bini Viotti, Stephanie M. Pouch, Maddalena Giannella, Monica Slavin, John W. Baddley, Ricardo M. La Hoz, Ligia Camera Pierrotti, Wanessa Trindade Clemente, Lilian M. Abbo

**Affiliations:** ^1^ University of Miami Miami Florida USA; ^2^ Jackson Health System Miami Florida USA; ^3^ Emory University Atlanta Georgia USA; ^4^ IRCCS Azienda Ospedaliero Universitaria di Bologna Bologna Italy; ^5^ Department of Medical and Surgical Sciences University of Bologna Bologna Italy; ^6^ University of Melbourne Melbourne Australia; ^7^ Johns Hopkins University School of Medicine Baltimore Maryland USA; ^8^ University of Texas Southwestern Medical Center Dallas Texas USA; ^9^ Universidade de São Paulo São Paulo Brazil; ^10^ Universidade Federal de Minas Gerais Belo Horizonte Brazil

**Keywords:** antibiotic prophylaxis, colonization, multidrug resistant bacteria, solid organ transplant, survey

## Abstract

**Background:**

Multidrug‐resistant organism colonization and infections cause significant morbidity and mortality in solid organ transplantation, affecting the perioperative antibiotic management. Yet, international practices for screening and antibiotic prophylaxis in colonized donors and recipients remain poorly defined.

**Methods:**

Self‐administered, web‐based survey conducted between February and July 2025 to assess global practices in multidrug‐resistant organism screening and perioperative antibiotic management in SOT, developed by transplant infectious diseases experts and endorsed by the Transplant Infectious Diseases Section of the Transplantation Society and the European Society of Clinical Microbiology and Infectious Diseases Study Group for Infections in Compromised Hosts. Data collected included respondent and institution characteristics; screening and prophylaxis protocols; donor and recipient colonization management; and timeframes relevant for prophylaxis modification.

**Results:**

Responses from 125 transplant centers across 24 countries and four continents were included. Most respondents were infectious disease physicians (73.6%). Antimicrobial stewardship programs and transplant infectious diseases consultation were available in 93.6% and 85.6% of centers, respectively. Over half (52.0%) modified prophylaxis based on donor multidrug‐resistant organism colonization, mainly triggered by urine and respiratory cultures. Preservation fluid and surveillance cultures influenced decisions less often. Recipient screening protocols were reported by 61.6% of centers, primarily targeting carbapenem‐resistant Enterobacterales (80.8%). About 41.6% routinely adjusted prophylaxis for colonized recipients, especially with recent (1–3 months) colonization.

**Conclusion:**

Substantial international variability exists in multidrug‐resistant organism screening and perioperative prophylaxis practices in solid organ transplantation. Evidence‐based consensus guidelines are needed to standardize and improve prevention of donor‐derived and recipient infections globally.

## Graphical abstract

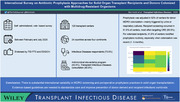

**Abbreviations**
ABTO: Brazilian Association of Organ Transplantation
AST: American Society of Transplantation
ASP: Antimicrobial stewardship program
CLSI: Clinical and Laboratory Standards Institute
CRE: Carbapenem‐resistant Enterobacterales
ESBL: Extended‐spectrum beta‐lactamase
ESCR‐E: Extended‐spectrum cephalosporin‐resistant Enterobacterales
ESGICH: European Study Group of Infections in Immunocompromised Hosts
EUCAST: European Committee on Antimicrobial Susceptibility Testing
ID: infectious diseases
IDCOP: infectious diseases community of practice
IQR: interquartile range
IRB: institutional Review Board
MDR GNR: Multidrug‐resistant gram‐negative bacilli
MDRO: Multidrug‐resistant organism
MDR‐E: Multidrug‐resistant Enterobacterales
MRSA: Methicillin‐resistant *Staphylococcus aureus*
OPO: Organ Procurement Organization
PAP: Perioperative antibiotic prophylaxis
PBP2a: Penicillin‐binding protein 2a
SOT: Solid organ transplant
TID: Transplant infectious diseases
TTS: The Transplantation Society
VRE: Vancomycin‐resistant Enterococci

## 1 Background

The prevalence of multidrug‐resistant organism (MDRO) colonization in solid organ transplant (SOT) candidates prior to transplantation varies by organism and transplant type, but pooled meta‐analyses estimate pretransplant colonization rates of approximately 8.5% for methicillin‐resistant *Staphylococcus aureus* (MRSA) and 11.9% for vancomycin‐resistant enterococci (VRE), with higher rates reported for multidrug‐resistant Enterobacterales (MDR‐E) in some cohorts (up to 25%–29%) [1, 2, 3]. Colonization rates tend to increase with longer pretransplant hospitalization and higher acuity of illness [4, 5].

Recipient MDRO colonization prior to transplantation is strongly associated with increased risk of posttransplant infection caused by the same pathogen [6, 7, 8] and has been associated with adverse outcomes and increased mortality [3, 4, 5, 9, 10, 11, 12, 13, 14, 15, 16, 17, 18, 19]. While direct cost estimates are not always reported, the increased infection rate typically leads to longer hospital stays, need for isolation, more expensive or prolonged antimicrobial therapy, and ultimately, increased healthcare costs and resource utilization in SOT populations.

Pre‐transplant screening for MDRO colonization in recipients has been proposed to guide targeted perioperative antibiotic prophylaxis (PAP) and infection prevention strategies. However, practices vary widely across transplant centers worldwide due to the lack of standardized guidelines and heterogeneous local epidemiology. Evidence guiding the optimal management of surgical prophylaxis in this setting remains limited, and while recipient colonization may warrant prophylaxis modification, the appropriate timing and extent of such changes are not well defined. In practice, the most common approach to screening SOT candidates for MDROs involves rectal swab cultures at the time of transplant to detect multidrug‐resistant (MDR) gram‐negative bacteria, including extended‐spectrum beta‐ lactamase (ESBL)‐producing organisms and carbapenem‐resistant Enterobacterales (CRE) [20]. Some centers also perform multi‐site sampling (e.g., rectal, nasal, and wound), particularly for kidney and liver transplant candidates [21].

Current international guidelines offer limited transplant‐specific recommendations for managing recipient MDRO colonization (Table 1). The American Society of Transplantation (AST) guidelines [22] suggest considering targeted screening for extended‐spectrum cephalosporin‐resistant Enterobacterales (ESCR‐E) and CRE in high‐risk settings, such as during outbreaks. However, AST does not explicitly recommend for or against modifying PAP based solely on ESCR‐E or CRE colonization. In contrast, European guidelines [23, 24] support active screening for MDR‐E, particularly in liver transplant recipients at centers with high prevalence, with subsequent adjustment of PAP for those who are colonized. They also recommend considering MDR‐E screening and targeted PAP for pancreas and small bowel transplant recipients.

**TABLE 1:** Summary of AST and European guideline recommendations for perioperative management of CRE, ESBL, MRSA, and VRE in solid organ transplant recipients.

Organism	American Society of Transplantation	European guidelines (ESCMID/ EUCIC)	References
ESBL‐producing organisms	Routine active surveillance remains undefined, particularly outside outbreaks. AST does not recommend for or against modifying PAP based solely on ESCR‐E colonization.	Recommend screening for liver transplant recipients in high‐burden settings; pancreas and small bowel recipients may also benefit. Recommend adjusting PAP in colonized liver recipients and considering targeted PAP for pancreas and intestinal recipients.	[22, 23, 24]
Carbapenem‐resistant Enterobacteriaceae (CRE)	Routine active surveillance remains undefined, particularly outside outbreaks. AST does not recommend for or against modifying PAP based solely on CRE colonization.	Recommend screening liver and intestinal recipients, especially in high‐prevalence centers. Recommend adjusting PAP in colonized liver recipients and considering targeted PAP for colonized pancreas and intestinal recipients.	[22, 24]
Methicillin‐resistant *Staphylococcus aureus* (MRSA)	Routine active surveillance is not recommended except in high‐prevalence settings. Consider adding vancomycin to PAP for MRSA‐colonized or previously infected SOT recipients, particularly cystic fibrosis patients undergoing lung transplantation, and patients with left‐ventricular assist device undergoing heart transplantation.	Active surveillance is recommended before high‐risk surgeries (e.g., cardiothoracic), although no transplant‐specific recommendations are provided. Adding vancomycin to standard prophylaxis is suggested for MRSA carriers undergoing cardiothoracic procedures, but there are no SOT‐specific recommendations.	[25, 26, 28]
Vancomycin‐resistant *Enterococci* (VRE)	No recommendations regarding active VRE surveillance or targeted PAP in colonized individuals.	Screening may be considered in liver recipients for epidemiologic or infection‐control purposes. Insufficient evidence for or against targeted PAP.	[27, 28]

Abbreviations: CRE, carbapenem‐resistant Enterobacterales; ESBL, extended‐spectrum beta‐lactamase; ESCR‐E, extended‐spectrum cephalosporin‐resistant Enterobacterales; MRSA, methicillin‐resistant *Staphylococcus aureus*; PAP, perioperative prophylaxis; SOT, solid organ transplantation; VRE, vancomycin‐resistant enterococci.

Regarding recipient MRSA colonization, the AST 2019 guidelines [25, 26] do not recommend routine active surveillance except in high‐prevalence settings. In colonized individuals—particularly cystic fibrosis patients undergoing lung transplantation and patients with left‐ventricular assist device undergoing heart transplantation—adding vancomycin to standard perioperative prophylaxis may be appropriate. In contrast, European guidelines do not provide transplant‐specific recommendations for MRSA.

Guidance for VRE is even more limited [26, 27]. AST guidelines provide no formal recommendations on routine screening or targeted PAP. European guidelines suggest that VRE screening may be considered in liver transplant recipients for epidemiologic or infection‐control purposes; however, evidence remains insufficient to support or refute the use of targeted PAP.

### 1.1 Donor Colonization and Implications for Transplant Outcomes

Similarly, beyond recipient screening, donor colonization and infection carry important implications for peri‐ and postoperative antibiotic management. Standard donor screening includes cultures from clinical sites such as blood, urine, and respiratory samples collected at the time of organ procurement. These cultures aim to detect donor active infection or colonization with MDROs to guide donor selection, recipient management, and post‐transplant prophylaxis [29, 30]. Risk factors for donor MDRO colonization include prolonged ICU stay, fever, prior antibiotic exposure, hepatitis C viremia, and prior hematopoietic cell transplantation [30, 31]. Although timely communication of microbiological results is critical, logistical challenges often delay result availability until after transplantation [32]. In practice, organs from colonized donors may still be used, but recipients should receive targeted antimicrobial prophylaxis and close post‐transplant monitoring [33]. Published guidance is available to assist in interpreting and managing positive donor cultures to prevent donor‐derived infections [34].

The lack of consensus regarding MDRO screening and prophylaxis in SOT candidates and donors is clinically important because it creates significant variability in clinical practice, which can lead to both under‐ and over‐treatment. Inconsistent screening protocols may miss MDRO colonization, increasing the risk of unrecognized transmission and post‐transplant MDRO infections, which are associated with higher morbidity and mortality in this population [3, 4, 5, 9, 10, 11, 12, 13, 14, 15, 16, 17, 18, 19]. Conversely, aggressive screening or broad application of targeted prophylaxis based solely on colonization status can lead to unnecessary use of broad‐spectrum antibiotics, increasing the risk of adverse drug events and promoting further antimicrobial resistance.

To address these gaps in practice and guidance, we conducted an international survey endorsed by the Transplant Infectious Diseases Section of the Transplantation Society (TID/TTS) and the European Study Group of Infections in Immunocompromised Hosts (ESGICH/ESCMID). We aimed to characterize global practices in MDRO screening and PAP in SOT, identifying areas of concordance and variation to inform future consensus guidelines.

## 2 Methods

This international survey assessed current practices regarding screening for MDRO colonization in SOT candidates and donors, as well as perioperative antibiotic management in colonized individuals undergoing transplantation. The questionnaire was developed by a multidisciplinary group of experts in transplant infectious diseases (TID) and pilot tested among four TGID experts for content clarity, validity, and reproducibility prior to dissemination. The survey was endorsed by TID/TTS and ESGICH/ESCMID.

The survey included 17 web‐based questions addressing: (a) respondent and institutional characteristics, (b) protocols for MDRO screening and surgical prophylaxis, (c) prophylaxis practices when the donor is colonized with an MDRO, (d) prophylaxis practices when the recipient is colonized with an MDRO, and (e) the time interval considered relevant when determining whether recipient colonization warrants modification of perioperative antibiotics (Supporting Information).

### 2.1 Survey Distribution

Between February to July 2025, the survey was broadly distributed to healthcare professionals involved in the care of SOT candidates and recipients. Invitations were sent via email through professional networks, including TID/TTS, ESGICH/ESCMID, the AST Infectious Diseases Community of Practice (IDCOP), and the Brazilian Association of Organ Transplantation (ABTO). The survey was also promoted on the TID/TTS Twitter/X account. Based on combined membership lists from these organizations, the survey reached an estimated 2986 professionals, although this figure is approximate due to substantial overlap in memberships and the possibility of multiple individuals from the same transplant center belonging to the same professional network.

The survey was self‐administered via Google Forms. Participation was voluntary, anonymous, without compensation, and did not involve the collection of personal identifiers or patient data. As no patient‐level data were collected and responses remained anonymous, ethics approval was not required, and the study was deemed exempt from formal institutional review board (IRB) review.

To minimize selection bias, the survey was disseminated broadly through multiple international transplant ID networks. Only one response per transplant center was included to avoid overrepresentation. In cases of duplicate responses from the same center, responses from ID specialists were prioritized, and if multiple ID specialists from the same institution responded, only the first submission was included.

### 2.2 Statistical Analysis

Descriptive statistics were used. Categorical variables are reported as frequencies and percentages, while continuous variables are presented as medians with interquartile ranges (IQR) and absolute ranges. No statistical comparisons were performed due to the exploratory nature of the study. Subgroup descriptive analysis was conducted by continent.

### 2.3 Definitions

MDR gram‐negative organisms were defined according to the modified criteria proposed by Rafaeilidis et al. [35]. ESBL‐producing bacteria were identified based on in vitro susceptibility, following the standards of either the Clinical and Laboratory Standards Institute (CLSI) [36] or the European Committee on Antimicrobial Susceptibility Testing (EUCAST) [37]. CRE were defined as isolates demonstrating in vitro non‐susceptibility to at least one carbapenem, based on CLSI or EUCAST breakpoints.

MRSA was defined as *S. aureus* resistant to oxacillin or cefoxitin, or harboring the *mecA* or *mecC* gene, which encodes an altered penicillin‐binding protein (PBP2a) with reduced affinity for beta‐lactam antibiotics [38]. VRE was defined as strains of *Enterococcus faecalis* or *faecium* with a vancomycin MIC ≥ 32 µg/mL or those carrying the *vanA* or *vanB* resistance genes detected by molecular methods.

Colonization was defined as the isolation of an MDRO from a rectal swab or other non‐sterile clinical sample (e.g., urinary, skin, or tracheal aspirate) in the absence of signs or symptoms of infection.

## 3 Results

**FIGURE 1:**
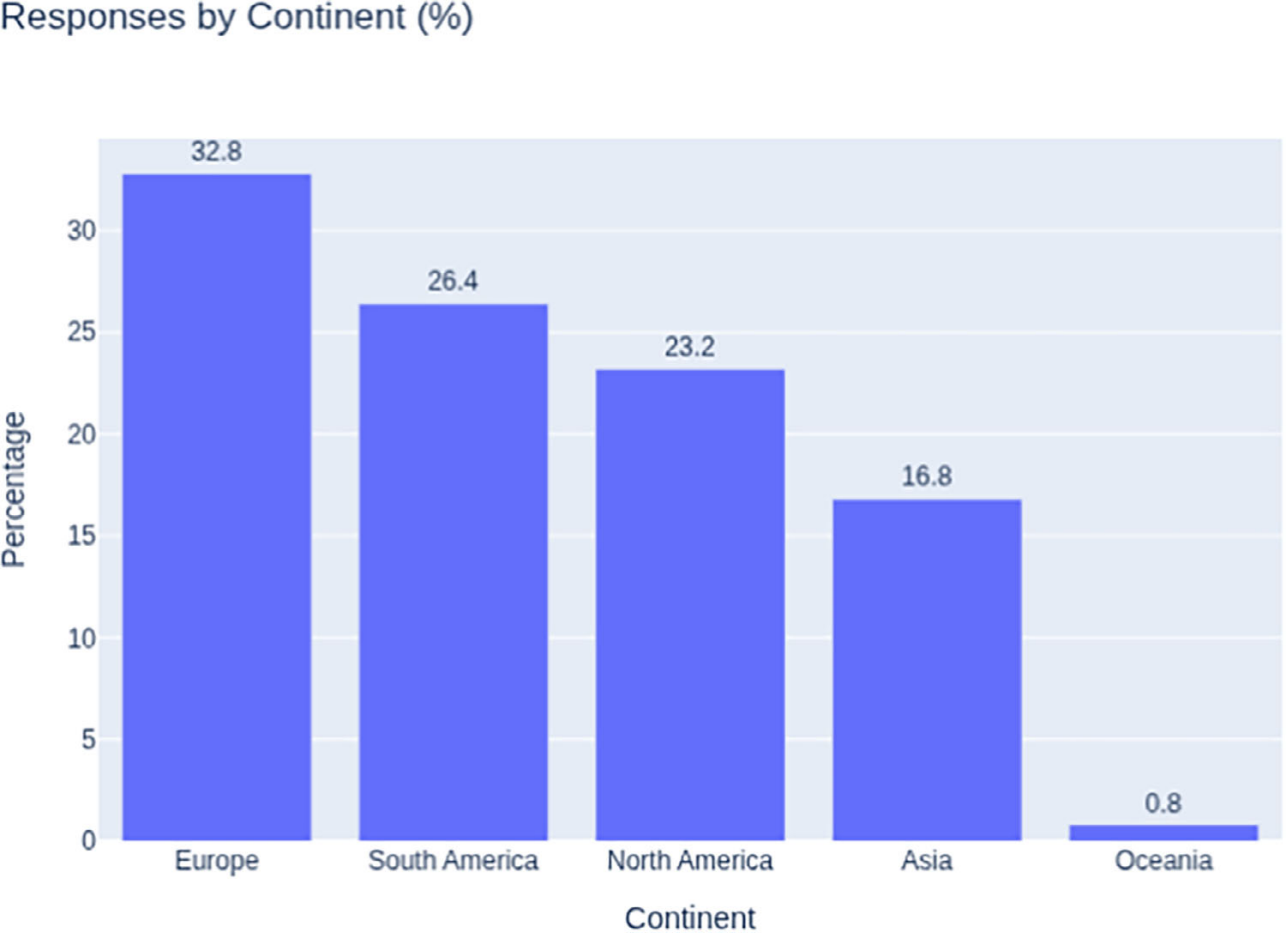
Survey responses by continent.

A total of 136 responses were collected across 24 countries and four continents. After excluding 11 duplicate responses from the same transplant centers, 125 unique responses were included in the final analysis. Most responses originated from Europe (41/125, 32.8%), followed by South America (33/125, 26.4%), North America (29/125, 23.2%), Asia (21/125, 16.8%), and Oceania (1/125, 0.8%) (Figure 1). No responses were received from the African continent. The top three represented countries were Brazil (30/125, 24.0%), the United States (29/125 responses, 23.2%), and Italy (13/125 responses, 10.4%).

Most centers reported performing kidney transplants (119/125, 95.2%), followed by liver (98/125, 78.4%), heart (81/125, 64.8%), liver‐kidney (66/125, 52.8%), lung (53/125, 42.4%), kidney‐pancreas (49/125, 39.2%), heart‐kidney (40/125, 32%), heart‐lung (30/125, 24%), multivisceral (19/125, 15.2%), intestinal (15/125, 12.0%), vascularized composite allograft (12/125, 9.6%), and other types of transplantation (4/125, 3.2%) (Supporting Information). Most respondents were infectious disease specialists (92/125, 73.6%), followed by advanced practice providers (10/125, 8.0%), transplant surgeons (9/125, 7.2%), infection preventionists (5/125, 4.0%), transplant hepatologists (3/125, 2.4%), transplant nephrologists (2/125, 1.6%), pharmacists (2/125, 1.6%), transplant pulmonologist (1/125, 0.8%), and one researcher (1/125, 0.8%).

**FIGURE 2:**
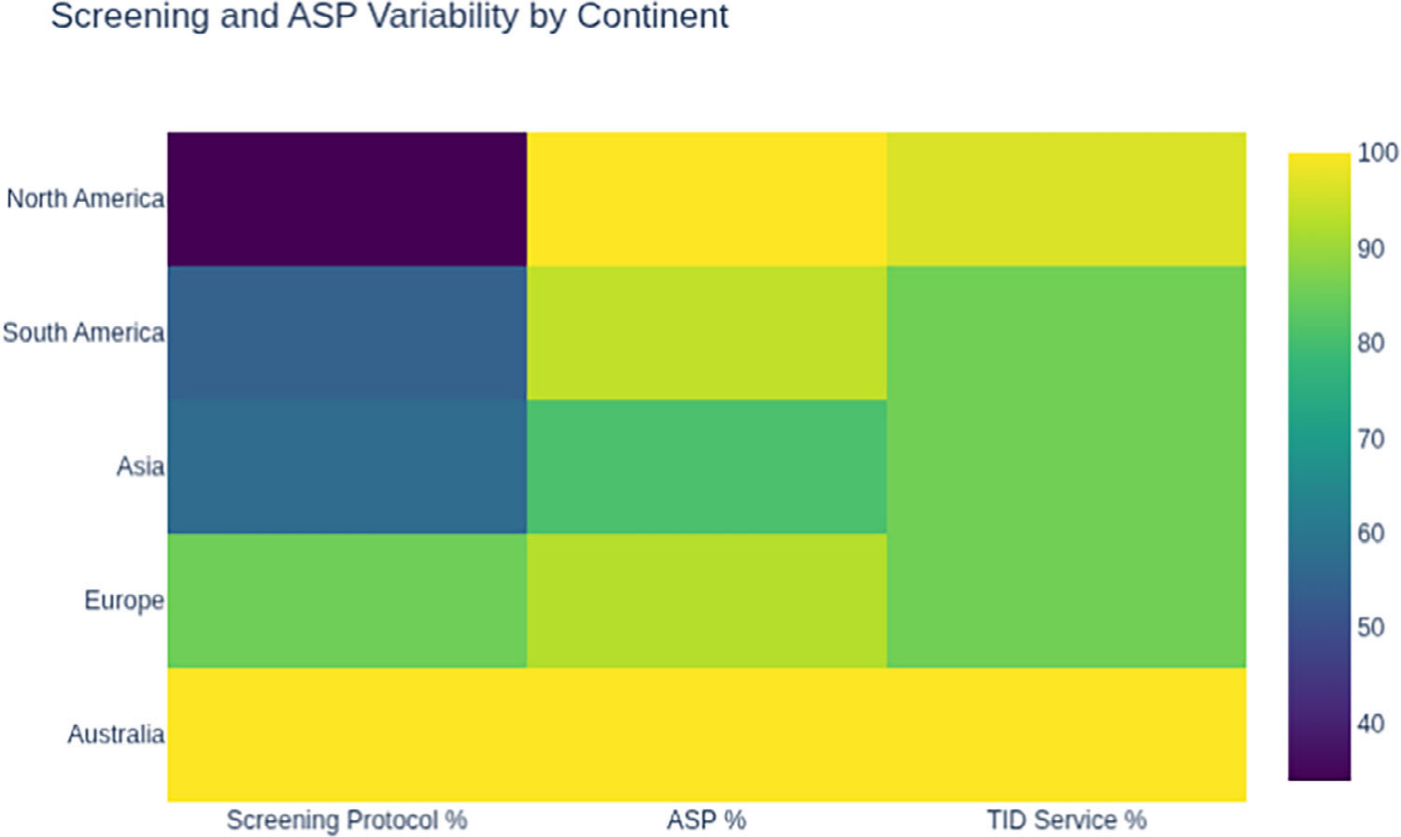
Screening and antimicrobial stewardship program variability by continent

An active antimicrobial stewardship program (ASP) was reported by 117/125 centers (93.6%). ASPs were reported in 100% of transplant centers in North America and Australia, 31/33 in South America (93.9%), 25/27 in Europe (92.6%), and 17/21 in Asia (80.9%) (Figure 2). A dedicated TID consultation service was available at 107/125 centers (85.6%), although access varied by continent, with the highest availability in Australia (1/1, 100%) and North America (28/29, 96.5%). However, only one center in Australia responded, limiting the interpretability of ASP and TID data for that continent. Access to donor microbiology data prior to transplantation was reported by 99/125 respondents (79.2%). Among these, 52/99 (52.5%) were directly notified by the organ procurement organization (OPO), 34/99 (34.5%) received results through an online platform, 9/99 (9.0%) obtained results through direct request (e.g., phone call), and 4/99 (4.0%) reported other methods.

Most hospitals (120/125, 96.0%) reported having a protocol for surgical prophylaxis in SOT recipients, with higher rates (97%–100%) observed in centers located in North America, South America, and Asia, and a lower rate in European transplant centers (87%). A protocol for screening MDRO colonization in transplant candidates was reported by 77/125 centers (61.6%) with notable variability across continents: Australia (1/1, 100%), Europe (35/41, 85.3%), Asia (14/21, 66.7%), South America (18/33, 54.5%), and North America (9/29, 31%). Among those with screening protocols, 62/77 (80.5%) screened for CRE, 48/77 (62.3%) for MRSA, 45/77 (58.4%) for VRE, 31/77 (40.3%) for ESBL‐producing organisms, and 21/77 (27.3%) for *Candida auris*.

### 3.1 Donor Colonization With MDRO

Among 125 respondents, 65/125 (52.0%) reported modifying standard surgical prophylaxis based on the donor`s colonization status, with notable variability across continents: Australia (1/1, 100%), Asia (13/21, 62%), North America (16/29, 55.2%), South America (18/33, 54%), and Europe (17/44, 38.6%). The most common indication for adjustment was MDROs detected in donor allograft cultures prior to transplantation (53/65, 81.5%), followed by colonization identified in donor cultures from sites other than the allograft (39/65, 60.0%) and from surveillance swabs or cultures (34/65, 52.3%). A smaller proportion (9/65, 13.8%) reported modifying prophylaxis in other scenarios. In contrast, 49/125 (39.2%) of respondents reported not altering prophylaxis in the setting of donor MDRO colonization, while 11/125 (8.8%) were unsure.

When specifically evaluating the approach to donors colonized with CRE, 60/125 (48.0%) reported adjusting surgical prophylaxis. Among them, most considered a donor urine culture positive for CRE in a kidney to be transplanted (54/60, 90.0%) and a respiratory culture positive for CRE in lungs to be transplanted (44/60, 73.3%) as indications to adjust prophylaxis. Adjustments were also reported for CRE‐positive cultures from preservation fluid (38/60, 63.0%), CRE identified in surveillance cultures (31/60, 51.6%), isolation from sites other than the allograft (29/60, 48.3%), and other circumstances (7/60, 11.6%).

Similarly, 57/125 respondents (45.6%) modified prophylaxis when donors were colonized with ESBL‐producing organisms, most commonly due to positive donor urine cultures in kidneys to be transplanted (38/57, 66.7%) and ESBL‐positive cultures from preservation fluid (29/57, 50.8%), with fewer respondents reporting changes based on respiratory cultures positive for ESBL in lungs to be transplanted (23/57, 40.4%), surveillance cultures (23/57, 40.4%), or colonization at other sites (20/57, 35.1%).

A comparable proportion (57/125, 45.6%) reported adjusting prophylaxis in the setting of donor MRSA colonization. In this group, 48/57 (84.2%) cited a donor urine culture positive for MRSA in a kidney to be transplanted as a reason for prophylaxis modification, followed by positive respiratory cultures in lungs to be transplanted (38/57, 66.7%), preservation fluid (32/57, 56.1%), colonization at other sites (28/57, 49.1%), and surveillance cultures (24/57, 42.1%). In addition, 8/57 (14.0%) indicated other scenarios.

Finally, 56/125 respondents (44.8%) modified surgical prophylaxis in response to donor VRE colonization, with the most frequently reported reasons being positive donor urine cultures (50/56, 89.3%), and isolation from preservation fluid (31/56, 55.4%), followed by positive respiratory cultures (29/56, 51.8%), colonization at other sites (24/56, 42.9%), and surveillance swabs (21/56, 37.5%).

**FIGURE 3:**
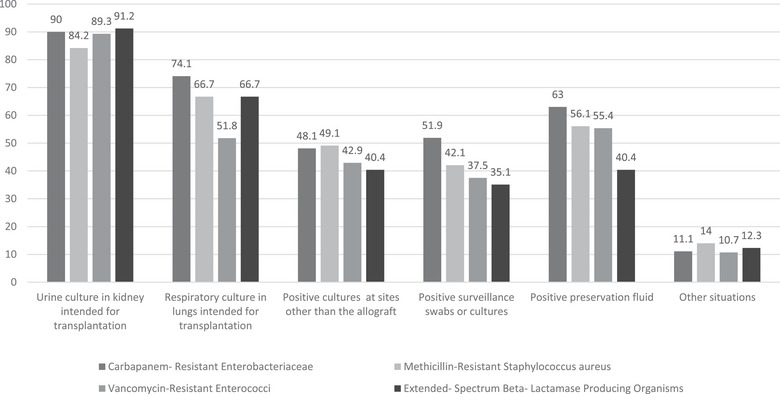
Modification of standard surgical prophylaxis based on donor's positive culture (%).

Overall, donor urine cultures from kidneys intended for transplantation were the most common triggers for modifying surgical prophylaxis across all MDRO categories (Figure 3). Donor respiratory culture from a lung intended for transplantation prompted antibiotic changes in up to 74% of cases, but were less likely to lead to modifications when donors were colonized with VRE. Although surveillance cultures were less frequently used than cultures from transplant‐relevant organs, a substantial proportion of respondents still considered them when adjusting prophylaxis, ranging from 20/57 (35.1%) for ESBL to 31/60 (51.9%) for CRE. Similarly, preservation fluid cultures influenced decisions for CRE (38/60, 63.0%) and MRSA (32/57, 56.1%), despite limited supporting evidence [39, 40, 41, 42, 43, 44, 45, 46].

### 3.2 Recipient Colonization With MDRO

Regarding the approach to recipients colonized with MDROs, 52/125 respondents (41.6%) reported routinely modifying standard surgical prophylaxis based on the recipient's colonization status, with notable variability across continents: Australia (1/1, 100%), Asia (11/21, 52%), South America (15/33, 45%), North America (11/29, 38%), and Europe (14/44, 31%). An additional 49/125 respondents (39.2%) indicated making such adjustments only in selected cases. Colonization with CRE was the most common trigger for modifying prophylaxis in both categories.

Among the 101/125 respondents who considered recipient colonization status when adjusting surgical prophylaxis, the most commonly cited time frames deemed clinically significant were 1–3 months (31/101, 30.7%) and less than 1 month (29/101, 28.8%) prior to transplant. Smaller proportions considered 3–6 months (13/101, 12.9%) or less than 1 year (16/101, 15.8%) relevant. In addition, 11/101 (10.8%) reported adjusting prophylaxis for any prior colonization, regardless of timing, while only 1/101 (1.0%) viewed colonization in the past 1–5 years as significant.

## 4 Discussion

This international survey provides insights into current gaps and practices surrounding screening for MDROs in both transplant recipients and donors, and how these practices influence perioperative antibiotic management in SOT. The considerable variation in screening protocols and in approaches to modifying surgical prophylaxis based on MDRO colonization aligns with prior survey findings in kidney and liver transplant recipients [21], highlighting significant heterogeneity in clinical practice worldwide. These discrepancies may reflect differences in local epidemiology, resource availability, institutional policies, or regional guidelines, emphasizing the need for context‐specific strategies to standardize best practices.

Encouragingly, the high prevalence (96%) of ASP reported by respondents represents a marked improvement over the past decade, underscoring the growing recognition of ASP's importance in the SOT population [47]. In a prior survey conducted in 2015, Seo et al. found that 74% of adult and 50% of pediatric SOT centers, respectively, had an institutional ASP [48]. This progress is further supported by widespread access to dedicated TID consultation, emphasizing the critical role of multidisciplinary expertise in tailoring perioperative management to local epidemiology and infection control priorities. Together, these resources form a foundation that can support more individualized and effective patient care.

Most centers reported having established protocols for MDRO screening in transplant candidates; however, there was notable variation between continents, with only 34% of North American centers reporting a protocol. CRE and MRSA were the most commonly targeted organisms, reflecting growing recognition of their clinical impact and consistent with studies showing that colonization often precedes post‐transplant infection [6, 7, 8]. In contrast, fewer centers reported screening for ESBL, VRE, and *Candida auris*, likely reflecting variability in local epidemiology, resource availability, or differing risk perceptions.

Our findings also revealed that more than half of respondents reported modifying surgical prophylaxis based on donor MDRO colonization, particularly when organisms were identified in urine or respiratory cultures from organs intended for transplantation. This aligns with current recommendations [34] and is clinically intuitive, given that colonization of the graft may increase the risk of donor‐derived infections. Notably, positive donor respiratory cultures for VRE were less likely to prompt antibiotic changes compared to other MDROs, possibly because VRE colonization in the respiratory tract may be perceived by clinicians as less likely a pathogen [49] and less clearly associated with adverse transplant outcomes.

Donor preservation fluid cultures influenced prophylaxis decisions in a smaller but significant number of transplant centers—particularly for CRE—despite mixed evidence regarding their predictive value for post‐transplant infections [39, 40, 41, 42, 43, 44, 45, 46, 47]. Across all pathogens, prophylaxis modifications were less common when donor preservation fluid was positive for ESBL compared with other MDROs. The clinical significance of positive preservation fluid cultures remains unclear, underscoring the need for further research to better define their role in guiding perioperative antimicrobial strategies.

Regarding recipient colonization, approximately 40% of respondents routinely adjusted perioperative antibiotics, and another 39.2% of respondents adjusted only in specific cases, with variation depending on the organism—22.4% to 39.2% for CRE and 13.6% to 30.4% for ESBL‐producing organisms. In contrast, a prior survey found higher rates of targeted PAP for recipients colonized with CRE (58%) and ESCR‐E (84%) [21]. These differing results highlight the ongoing heterogeneity in clinical practice. The time interval deemed relevant for prophylaxis modification also varied; most centers considered colonization within 1–3 months before transplant as most significant, contrasting with the earlier survey [21], which suggested a window of up to 3–6 months.

Our study has limitations consistent with survey‐based research, including potential response bias, reliance on self‐reported data, and underrepresentation of certain geographic regions—particularly Africa and Oceania. In contrast, some areas, such as Brazil and the United States, were heavily represented, which may have skewed the findings. In addition, kidney and liver transplant programs were overrepresented, potentially limiting the generalizability of results to centers performing other types of organ transplants. Despite these limitations, the international scope and multidisciplinary respondent pool offer valuable baseline data to inform future research and guideline development.

In conclusion, this study highlights the wide global variability in MDRO screening and perioperative antibiotic practices in SOT. The absence of standardized protocols reflects evolving local epidemiology as well as critical gaps in high‐quality evidence to guide clinical practice. There is a pressing need for randomized controlled trials to evaluate the effectiveness and safety of different MDRO screening strategies and tailored PAP regimens. Such studies should incorporate standardized definitions of MDRO colonization, core outcome sets, and integration with existing transplant registries to ensure comparability and generalizability of findings. Endpoints should include post‐transplant MDRO infection rates, graft survival, patient morbidity and mortality, antibiotic‐related adverse events, costs, and the impact on antimicrobial resistance patterns. Such robust data are essential to develop evidence‐based guidelines that optimize infection prevention, improve transplant outcomes, and support antimicrobial stewardship efforts. Given the ongoing challenges posed by antimicrobial resistance and the limited availability of donor organs, it is essential to establish optimized, evidence‐based, and cost‐effective antibiotic prophylaxis strategies that effectively prevent infections while preserving antibiotic efficacy and ensuring the best possible outcomes for transplant recipients.

## Conflicts of Interest

The authors declare no conflicts of interest.

## Supporting information




**Supporting File 1**: tid70154‐sup‐0001‐SuppMat1.docx

**Supporting File 2**: tid70154‐sup‐0002‐SuppMat2.docx.
