# Supplementary figures and images for: International Survey on Antibiotic Prophylaxis Approaches for Solid Organ Transplant Recipients and Donors Colonized With Multidrug‐Resistant Organisms
Source: Transpl Infect Dis. 2025 Dec 16;28(1):e70154. doi: 10.1111/tid.70154 (PMC12892828; doi:10.1111/tid.70154)

**Supplementary Material 1 :** Electronic Survey Questionnaire.


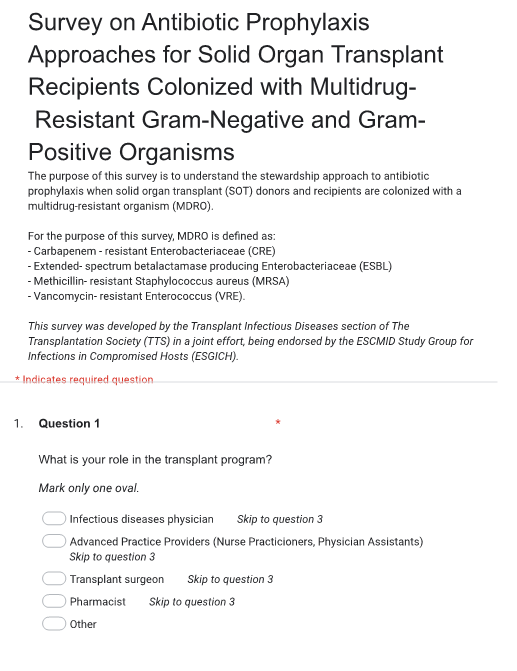


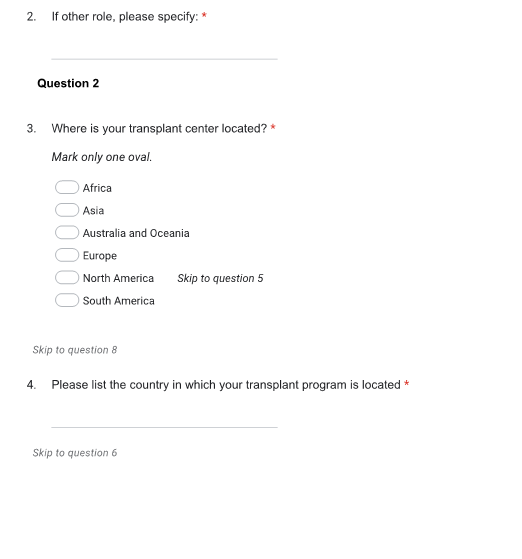


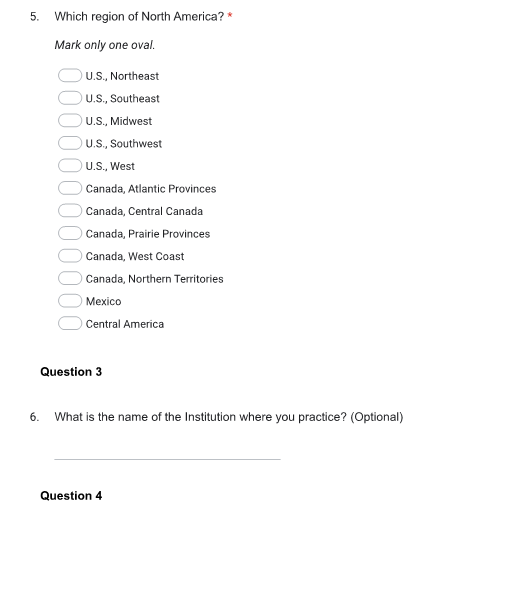


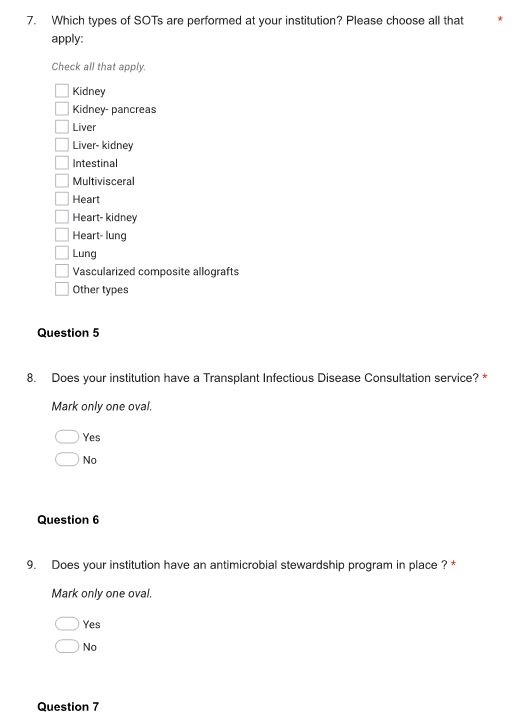


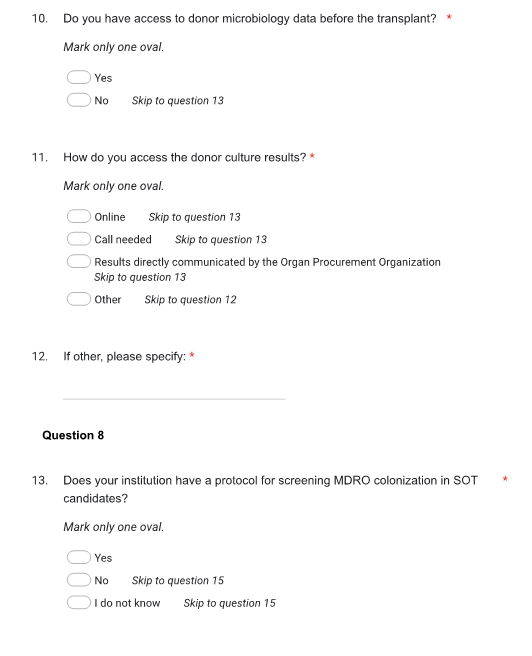


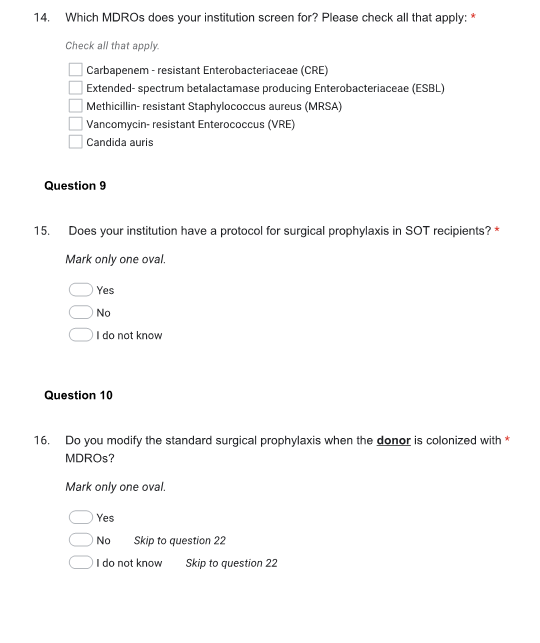


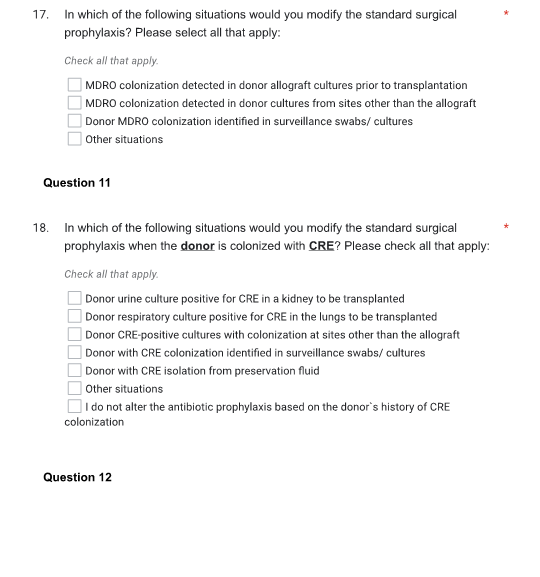


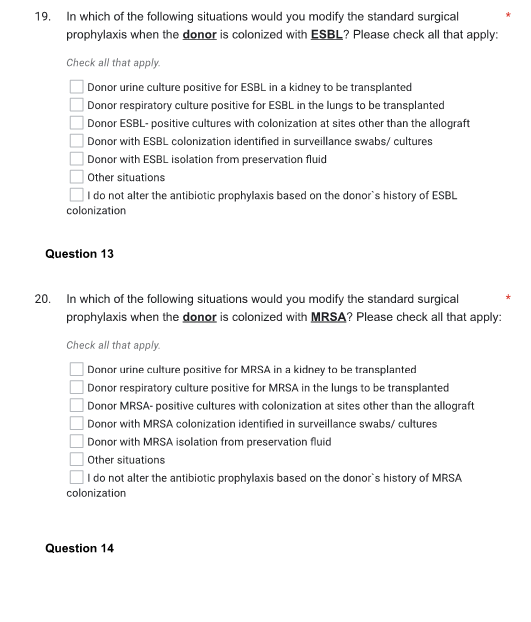


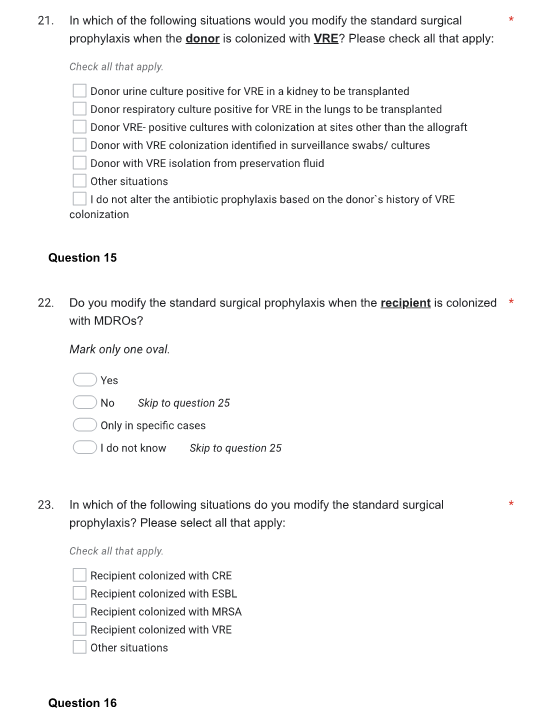


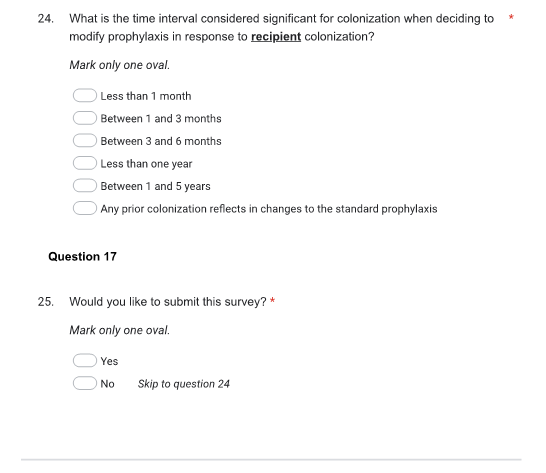

Supplement: Supplementary file 1 — Supporting File 1: tid70154‐sup‐0001‐SuppMat1.docx [file TID-28-e70154-s001.docx]

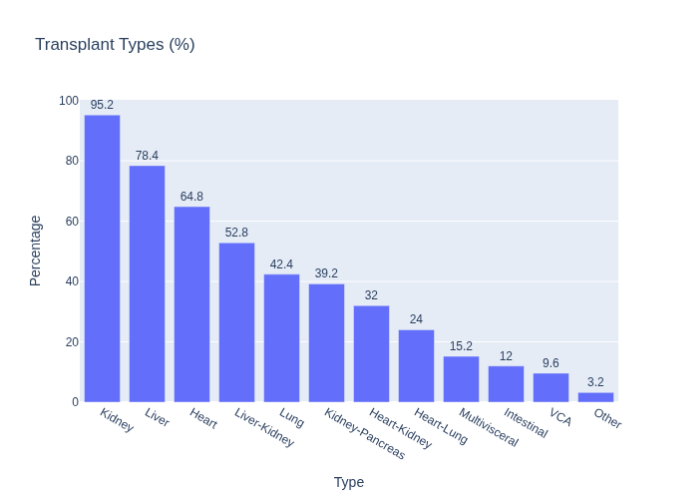

**Supplemental Material 2:** Transplant types

Supplement: Supplementary file 2 — Supporting File 2: tid70154‐sup‐0002‐SuppMat2.docx. [file TID-28-e70154-s002.docx]
